# On the Presence of Air in the Veins as a Cause of Death

**Published:** 1864-02

**Authors:** Jas. Sumner Greene

**Affiliations:** Dorchester, Mass.


### SELECTED.

### ON THE PRESENCE OF AIR IN THE VEINS AS A CAUSE OF DEATH.

By JAS. SUMNER GREENE, M. D.,

*Of Dorchester, Mass.*

Our attention was recently called to this subject under circumstances which led us to consult authorities for the purpose of ascertaining the supposed frequency of the accident, the conditions under which it may occur, the signs and symptoms attending it, and the nature and amount of evidence required in a given case of death to prove that it resulted from this cause.

These inquiries satisfied us: 1st, That medical gentlemen generally entertain but vague ideas on the subject; 2d, That there is plenty of evidence on record of the occurrence of the accident often enough, under various circumstances, to make it requisite that every practitioner of medicine and surgery should be sufficiently aware of the character of the danger to be able to take ordinary precautions for its prevention, to recognize it when it occurs, and to act promptly and understandingly for its relief; and, 3d, That the investigations heretofore made, while they throw much light on the subject, are far from being complete and satisfactory; that the occurrence of the accident is at least possible under circumstances which have never yet been discussed in this light; and that the interests of science and humanity require, further, more careful and systematic experiments and observation to settle the questions in dispute and clear up the points of obscurity.

Convinced of these things, our object in this paper will be, after some observations illustrative of the first mentioned point, to present as concise a view as possible of all the facts which we have obtained, indicating their chief sources. But while showing what is known, we hope at the same time to throw some new light on the subject, and to indicate what ought to be further elucidated; thus furnishing an index for future reference, and a starting point for new investigations.

There are several reasons why the subject is not better understood among us. In the first place, in its practical bearings, it is a comparatively recent one. Sufficient care has not always been taken, in making experiments, to guard against sources of fallacy, and opposite and contradictory theories have been proposed to account for the phenomena noticed. Moreover, many of the reports of accidental death referred to this cause have been meagre and unsatisfactory; while from the nature of the symptoms—they being such as may occur under similar circumstances, but from other causes than the one in question—there is room for doubt as to the agency; and against a majority of the cases on record, objections on one or the other of these grounds might be raised with considerable plausibility. For these reasons the subject has not yet fairly taken rank among the unquestioned facts of medical science, and is very unsatisfactorily treated by our latest standard authorities on surgery, obstetrics, pathology, and medical jurisprudence.

For example, none of the works on morbid anatomy which we have consulted make any allusion to the subject, except that of Jones and Sieveking (p. 364,) and their remarks are comprised in a single paragraph. Of the works on medical jurisprudence in common use, only that of Taylor (p. 271) mentions the subject; while he refers to the accident only as connected with wounds about the neck, and makes statements as to its nature and causes which reveal very imperfect knowledge of the matter. Its surgical aspect is that best understood, yet Druitt dismisses it in twelve lines (p. 584); while Erichsen, who alone gives it due prominence, treats it as he has elsewhere, ably but somewhat controversially (p. 140), as do many of the French surgical authorities. Finally, Churchill's is the only system of obstetrics which makes any allusion to it whatever (p. 428). His remarks are just and valuable, but refer only to the occurrence of the accident after delivery, leaving other circumstances of not less importance unremarked. And yet, as will appear hereafter, the subject has important practical bearings upon each of these branches.

We will first consider the accident as it occurs during surgical operations, to which class a majority of the cases now known belong, and for convenience we shall include certain suicidal cases in the same category.

The first well authenticated instance occurred in the year 1818, in the hands of M. Beauchêne, at the Hôpital St. Antoine (Ollivier, *Dict. de Méd.*, art. "Air"). Within a few years thereafter similar cases were reported by MM. Dupuytren, Castara, Delpech, Roux, and Ulrich (Velpeau, *Leçons Clinicales*, vol. i. p. 451, et seq., Amussat, *Recherches sur*

*l'introduction accidentelle de l'air dans les veines*, and Ollivier, *loc. cit.*,) all ending fatally, and verified by post-mortem examination. Numerous other cases were made known, in which the symptoms bore close analogy to those before named, but which ended in recovery. Prominent among these are cases by MM. Roux, Clémot, Goulard, Mirault, Rigaud, Delaporte, Malgaigne, and Amussat (*Id.*). In this country a case was reported by Dr. Valentine Mott (*Am. Journ. Med. Sci.*, Nov. 1828, p. 107), one by Dr. Mussey (*Id.*, Feb., 1838, p. 391), and three by Dr. John C. Warren ("On Tumours," 1839, and *American Cyclopædia of Pract. Med. and Surg.*, vol. i. p. 263). Two of Dr. Warren's cases were well marked, and though recovery followed in one of them, and in the other no autopsy was obtained, yet no doubt as to their nature existed in the minds of the surgeons who witnessed them. In 1838, M. Velpeau took up the subject in his *Cliniques* (*op. cit.*,) giving a synopsis and brief analysis of 31 cases, including those of Drs. Warren and Mott, and one by himself. The following year M. Amussat's work appeared (*op. cit.*,) in which the details of thirty-six cases occurring in human beings were given. In 1843, Professor Wattman, of Vienna, published a monograph on the subject, in which he reported four cases witnessed by himself. Not having seen his paper, we derive our knowledge of it from able reviews by Dr. John Reid (*Physiological Researches*, p. 539), by a writer in the *Am. Journ. Med. Sci.*, vol. ix. p. 170, and by one in the *Br. and For. Med. Rev.*, vol. xxiii. p. 443. The last named writer collected and arranged in a tabular form 54 cases, more or less authentic and complete in detail, including all those of MM. Velpeau and Amussat, except four very doubtful ones of the latter, which occurred before the possibility of the spontaneous entrance of air into the veins was recognized. Besides these we find cases reported by Dr. March, of Albany, (COOPER's *Surg. Dict.*, 1838), by Dr. Marcacci (*Revue Médicale*, 1847, p. 599), by Prof. Portal, of Palermo (*Id.*, 1839, p. 98), by M. Riberi, of Turin (*Gazette Medicale*, 1843, p. 204), by M. Schmid (*Id.*, 1851, p. 561), by M. Girbal (*Id.*, 1853, p. 46), by Dr. R. H. Coolidge, U. S. A. (*Philadelphia Medical Examiner*, 1849), by Dr. Willis, of Barnes, England (*London Med. Gaz.*, xli. p. 608), by Mr. G. F. Lane (*Id.*, xlv. p. 926), and by Mr. Ward and Mr. Hutchinson, each a case (*Medical Times and Gazette*, Feb. 1856). That reported by Dr. Coolidge was a case of suicide. Finally, and while this paper is undergoing revision for the printer, we are apprised of another accident of a similar nature occuring under the hands of Dr. Henry G. Clark, of Boston, Surgeon at the Mass. General Hospital.* It is very possible that other cases have been reported, but these are all that have met our observation.† Of these 67 cases, much the larger portion occurred during the removal of tumors of the neck, breast, and axilla; two, those of B. Cooper and Delpech, were in amputations at the shoulder-joint; one, that of Mussey, was during amputation of the scapula and clavicle; two, those of Rigaud and Clémot, were while tying the subclavian artery; three were during venesection at the external jugular; three during venesection at the median vein; and one, that of Willis, was during the insertion of a seton needle in the neck. Among the veins wounded were the external jugular in 13 cases, including the three above named; the internal jugular in 10 cases, the subclavian in the case of Mussey and the axillary in that of Goulard. One, attributed to Dupuytren, is referred to the internal saphena vein; but this, as well as those referred to the median vein, is not entitled to much confidence. In the others the vessel was either not known, or was ascertained to be a branch of one of the above named veins, cut either about the neck, shoulder, axilla, or breast.

What are the characteristic symptoms? During the progress of an operation, when all seems going on well, a peculiar sound is heard at the bottom of the wound, oftenest described as gurgling, hissing, or bubbling. There is frequently a slight issue of venous blood, indicating that a vein is wounded, and often bubbles of air are noticed at the point from whence the sound proceeds, as in the cases of Drs. Warren Mussey, Clark, and others. The patient suddenly turns pale,

* The patient in Dr. Clark's case was undergoing an operation for the removal of a very large glandular tumor, occupying the whole side of the neck, and during the latter part of it, and when traction was made upon the tumor, a sucking sound was heard, and bubbtes were distinctly seen to enter the jugular vein near the clavicle. The vein had been tied above the wound when the operation was begun, and therefore there was no bleeding to obstruct the view of the orifice. Pressure was instantly applied, and the sound ceased. The vein was then tied below the wound, and the operation was completed without any apparent ill effects. These facts were kindly furnished us by Dr. Clark, who will publish a detailed account of the case in the *Transactions of the Boston Society for Medical Improvement.*

† Mr. Taylor refers to a case published in the *Association Journal* (1853, p. 9), but we have not the particulars. Prof. Valentine Mott writes to M. Amussat (see Amussat, *op. cit.*, p. 157) that he has witnessed three fatal cases in this country; but the details he gives do not enable us to decide whether they are identical with any of those included in the above category or not.

utters a cry, such as "I am faint," "I am dying," and becomes insensible; or there may be observed anxiety of countenance, labored respiration, lividity of the lips, dilated pupils, and convulsions. These symptoms may gradually give way and the patient recover, or they may result in death. The state of the pulse is very apt to be overlooked in the confusion incident to such dangerous and unlooked for symptoms; when sought, it is usually feeble and often imperceptible. Sometimes, however, as in the case of Dr. Mott, violent and irregular action of the heart is observed. Again, lividity of the face and stertorous breathing may be prominent symptoms, as in the cases of Dr. Warren. In the cases of Drs. Beauchêne, Mussey, Portal, and Girbal, a cold sweat was seen to break out upon the face. In Dr. Warren's fatal case a vermillion flush was seen upon the previously livid cheeks during the progress of the efforts at restoration, but soon vanished. In the case of Marcacci a violent cough, lasting twenty minutes, was a noticeable symptom. In Bransby Cooper's case there was involuntary ejection of urine and feces. Syncope is often the predominant feature, and the patient may die with scarcely a struggle. A dull, rustling sound was heard in the thorax in one of Wattman's cases, distinct from the hissing noise at the wound; and the same sound is said to have been heard in Beauchêne's case, but we do not find it mentioned in any of the accounts of it which we have consulted.* In the later case, by M. Girbal, auscultation of the heart was practised, and revealed a *bruit de gargouillement*, completely masking the tic-tac. These, however, are the exceptional signs and symptoms; usually their course is much as at first described. A few of the more prominent ones require further notice.

The warning sound at the wound is almost invariable. Thus it was distinctly noticed and described in 48 instances, including one of suicide, and comprising almost all the cases of which we have any details. It has been described as a peculiar sound (*un bruit particulier*) exactly like that which air produces in penetrating by a small opening into the chest of a living animal, a prolonged hissing (*un sifflement prolongé*) analogous to that produced by the return of air into an exhausted receiver (*par la rentrée de l'air dans un recipient où l'on a fait le vide*), a very noisy snuffling (*reniflement très bruyant*), &c., &c., (Ollivier, *op. cit.*). It is sometimes re-

* See Ollivier, Velpeau, and Amussat, *op. cit.*

peated more than once, as in the cases of Castara, Rigaud, and Clémot. It always ceases upon placing the finger upon the spot whence it issues, and is likely to be renewed if the pressure is removed, as occurred in the cases of Beauchêne and Clémot, and in one of Wattman's.

Directly upon this sound usually follow syncope or convulsions, or both. In 25 cases, only the symptoms of syncope were remarked; in 6, convulsions without syncope; while in 7, syncope was succeeded by convulsive spasms. In the remaining cases, either no general symptoms occurred or death was described as immediate, and no particulars given. Tetanic spasm was noticed in the case of Mirault; opisthotonos, in that of Asmus; and distortion of the face, followed by temporary hemiplegia, in that of Mott. In 13 cases, the respiration was noticed to be embarrassed, hurried, or irregular.

A sudden cry or exclamation was heard in 17 cases. "My blood is falling into my heart; I am a dead man," were the words uttered by Beauchêne's patient. In the case described by Mr. Lane, a small opening was made in a tributary of the axillary vein, a noise like the sucking of water and air into a syringe was heard, and the patient exhibited symptoms of syncope, which were followed by convulsions. She recovered, and in describing her sensations afterwards, said that "she felt something bubble under her arm and shoot quite cold across her breast, and just as she went off she got quite cold all over." This symptom is of course wanting when the patient is fully etherized, as in Dr. Clark's case. In M. Girbal's, the patient had inhaled chloroform, but was becoming conscious at the time of the accident, and uttered a "slight, plaintive cry." Nine cases in which the sound was heard were followed by no bad consequences whatever, and 24 recovered after symptoms of more or less severity. Of the fatal cases, 24 died immediately, or within a few minutes after the conclusion of the operation. The patient of Willis lived 7 hours; that of Mirault, 3 hours; that of Clémot, some hours; that of Girbal, 13 hours; one of Roux, 7 days; and one of Wattman's, 28 days. Of the remaining 4 cases, we have no particulars. The patient of Wattman died finally of pneumonia; that of Girbal, with symptoms of asphyxia; but no autopsy was made in either case.

Of the fatal cases, 18 were examined after death. In that of Graafe it was merely reported that no important organ was wounded, and that the heart and great vessels were natural.

It was mere conjecture that placed it in the present catalogue; all that was stated of the circumstances being that syncope and death occurred during the removal of a gland in the axilla. In the case of M. Beauchêne neither air nor blood was found in any of the cavities of the heart; but there were numerous bubbles of air in all the vessels of the brain, in the inferior vena cava, iliac veins, aorta, and crural arteries. Dissection in this case was made eighteen hours after death. In the case of M. Roux, where death was delayed until the seventh day, the cavities of the heart were also found empty, and no air was discovered in the veins, while the aorta and iliac arteries contained air mixed with blood. It is noticeable also that the lungs were œdematous, and the bronchia contained frothy mucus. In the remaining 15 cases air was found in the right cavities of the heart. In 4 cases, besides the two above named, air was found in the arterial system: those, viz., of Dupuytren and Gorré, and the suicidal cases of Pellis and Handyside. In 6 cases the air in the heart is stated to have been mixed with blood; in 2 it is stated that it was not so mixed; while in the remainder the condition in which it existed was not remarked. In 8 cases air was noticed in the venous system; in only 2, those of Willis and Coolidge, was it particularly noticed in the pulmonary artery. In both cases of M. Roux it was stated that no air was found in the vessels of the brain; while in M. Dupuytren's case it was found there. The length of time elapsing before the autopsy, is stated in but six of the cases: in these it was from 18 to 26 hours, with the exception of one, in which it was 52 hours. In the case of Roux a rough analysis of the gas contained in the heart was made, and it was decided to be atmospheric air. In the case of Pellis an analysis was made by M. Bischoff with a similar result. M. Roux also measured such of the air contained in the right ventricle as he could collect, and found 11 centimetres cubes (about two-thirds of a cubic inch).

We have now seen that accidents, all dangerous, and many fatal, do occur under specified circumstances, ushered in by a sound indicating the entrance of air into a vessel, attended by a peculiar class of symptoms, and in which the autopsy, if one is made, uniformly discloses the presence of air in the circulatory system. Let us now see if anything is known corroborative or explanatory of these facts. Here, as in all physiological investigations, we resort to experiments upon animals. We find, however, that experiments bearing upon the subject were made long before the subject itself was known to have any practical importance.

Nearly two centuries ago it is stated that Wepfer killed an immense ox by blowing air into the jugular vein. Similar experiments were afterwards made upon dogs and sheep by Redi, Bohn, Heydius, R. J. Camerarius, Brunner, Harder, Sprœgel, and Valisneri (MORGAGNI, *De Sed. et Caus. Morb.*, Epis. v.). Dr. Langrish, an English physician of the last century, made similar experiments upon dogs; and Châbert, a French *veterinaire*, upon horses. The chief practical result seems to have been the adoption of this as a convenient mode of killing superannuated horses (OLLIVIER, *op. cit.*, p. 66). At the beginning of the present century interest was reawakened in the subject by Bichât, who made some new, but as it proved, erroneous statements relative to the amount of air necessary to produce death, believing that a single bubble alone was sufficient (*Récherches Physiol. sur la Vie et la Mort*, art. ii. part ii.). In 1809, Nysten published the results of numerous and careful experiments made by him upon animals with a view to determine the effects produced upon the animal economy, by the presence of atmospheric air and various gases in the circulatory system. At this time nothing was known of the possibility of the spontaneous entrance of air into an opened vein, except a case reported in 1806 by Verrier of its entrance into the jugular vein of a horse when opened in phlebotomy (VELPEAU, *op. cit.*). It is true that Redi and Caldesi saw air circulating in the veins of tortoises, Lancisi in hedgehogs, and Morgagni in vipers, trout, and carp, after being wounded; but they inferred that it existed naturally in those animals. It is true also that Méry noticed the appearance of air in the lower vena cava of a dog, after puncturing the vein; but he thought it was developed in the smaller venous branches, and passed forward to fill the vacuum caused by the escape of blood (MORGAGNI, *op. cit.*, secs. 22–26). Haller, indeed, who witnessed the same phenomena, was convinced that they only appear after a considerable wound of some vessel, and that they are not seen when the veins have been well managed. Nysten himself had observed many times that the veins and right auricles of decapitated men were distended with air; but he made no attempt at its explanation (*Récherches de Physiol. et de Chimie Pathologique*, p. 5). After instances became known of death in the human species attributed to this cause, these experiments and observations became matters of more practical interest. Dr. Wing, of Boston, published the result of experiments made by him upon rabbits and sheep (*Boston Med. & Surg. Journ.*, May

14, 1834). Other experiments are recorded by Magendie (*Leçons sur les Phénomènes Physiques de la Vie*, 1836), and by Dr. John Rose Cormack (*Inaugural Dissertation*, 1837). In 1837 a warm discussion arose in the French Academy of Medicine, consequent upon the account given by M. Amussat of a case occurring under his hands, and where recovery was attributed to the means used by him (*Bulletin de l'Academie*, vol. i. pp. 895 and 905). On this occasion M. Gerdy and others denied the probability of the entrance of air causing death in human beings, chiefly on the ground that the symptoms reported differed from those noticed in animals when air was forcibly injected. The discussion resulted in the appointment of a commission, of which M. Bouillaud was reporter, before which M. Amussat exhibited a series of forty experiments on dogs, horses, and mules, to determine the effects of the spontaneous entrance of air, and the various circumstances which modify those effects (*Id.*, vol. ii. p. 182). Later experiments have also been recorded by Mr. Erichsen (*Edinburgh Med. and Surg. Journ.*, Jan., 1844,) by M. Bernard) *Des Substances Toxiques et Médicamenteuses*, pp. 161–163), and by Prof. Dalton (*Am. Med. Monthly*, June, 1860, p. 507).

Some of these results are in brief, as follows: It was found that the injection of air into the veins of a living animal, whether by means of a syringe, as in the experiments of Nysten, or by insufflation through a tube, as in those of Dr. Cormack, produced symptoms proportioned to the quanty used and the rapidity with which it was injected. When injected little by little, and repeatedly, the result was the expectoration of a transparent frothy liquid, mucus râles, and death sometimes as late as the third day. A still less quantity produced only momentary excitement of the heart's action, followed, after repeating the injection, by an enfeebling of the pulse. Sometimes complete recovery took place after quite violent symptoms. The phenomena produced in the fatal cases were as follows: A *bruit particulier* was heard, coming from the region of the heart, and synchronous with its systole. This was caused by the mixture of air and blood taking place in that organ, and sometimes lasted many minutes. It was compared to the sound produced by beating white of egg and water together (*en battant ensemble du blanc d'œuf et de l'eau.* Nysten, *op. cit.*, p. 16). The animal uttered some cries of suffering after the first injection, the pulse became more frequent, the respiration quickened and panting, the limbs stiffened and sometimes agitated by convulsive movements, the urine and fecal matters were forcibly expelled, and in proportion as the experiment was prolonged the pulse grew feeble and ceased to be felt, the inspirations became rare and deep, and death soon followed. If a fatal quantity was introduced at a single injection the effects were sudden falling, cries, convulsions, and almost instant death. Dr. Wing noticed in some of his cases that the heart palpitated violently, and then suddenly ceased altogether. We have noticed the same thing in a cat subjected to a like experiment. The heart thumped violently against the walls of the chest several times, ceased suddenly, and again thumped as before. Its action ceased altogether in less than a minute after air entered, with the exception of the right auricle, which continued its contractions for several minutes.

The quantity of air necessary to produce death varied with the different species of animals, and among different individuals of the same species. Some of the earlier experimenters noticed that dogs succumbed sooner than sheep, and Morgagni concluded that it was because the latter were colder blooded animals (*op. cit.*, sec. 22). Nysten estimated that between 40 and 50 centimetres cubes (2.44 to 3.05 cubic inches) for a small dog, and from 100 to 120 centimetres cubes (6.10 to 7.32 cubic inches) for a large dog were required, and much larger quantities for larger animals. Dr. Wing found that a volume equal to f℥ iij did not kill sheep; the symptoms subsided in about 20 minutes—f℥ vj produced apparent death in a sheep, and f℥ iij killed rabbits in a few seconds. According to Mr. Rey, Professor in the Veterinary School at Lyons, two full expirations are required to kill horses, and sometimes a much larger quantity fails to cause death (*Gaz. Hebdomadaire*, 1861, p. 437). He states that he has heard the *bruit de glouglou* in these animals for some hours after bleeding without any accompanying disturbance of the general functions.

The experiments of the French commission, and those of Erichsen, are the only ones recorded in which the air was spontaneously admitted; in these the circumstances were made to resemble, as closely as possible, those under which it accidentally enters the veins in man. The vein opened was the internal jugular in most of the cases; once the subclavian was selected, once the axillary, and once the brachial near the border of the axilla. It was found that air always entered these veins in those parts to which the venous pulse extended, and not beyond that limit unless the orifice was put upon the stretch, or a tube inserted to the part where the venous pulse existed. The entrance of air was always announced by a lapping sound (*lappement*) in dogs, and a gurgling (*glouglou*) in horses. This was heard at the wound, and was generally synchronous with the inspiration, but sometimes with the diastole of the heart. Auscultation of the heart in dogs revealed a sound described as a mixture of blowing and gurgling (*un mélange de souffle et de glouglou*) as a churning sound, or as a *bruit de souffle* accompanied by a *bruit humide*. This sound is not mentioned as occuring in horses. During expiration frothy blood often made its escape from the wound. The symptoms were much as when air was injected, but death was more gradual. Anxiety and agitation, great debility, labored respiration, cries, and violent convulsions, with ejection of urine and feces, preceded the fatal termination. Death occurred in from 3 to 36 minutes in healthy dogs. It took place sooner in the vertical position than in the horizontal. The symptoms seemed to be aggravated in this position, probably in part because the escape of blood and froth at the orifice in the vein was prevented. It was found that death took place sooner if the animals were debilitated before the experiments. A few experiments were tried to ascertain the remedial effects of suction and of compression of the chest, but they gave no satisfactory results.

Examination after death constantly showed great distension of the heart with frothy blood, which was also found in the pulmonary artery, and in more or less of the larger veins. In horses, air was also found in the arterial system in all cases.

This was attributed by M. Bouillaud to the larger size of the pulmonary capillaries in those animals than in others, an explanation which may also help to account for the greater quantity of air required to produce fatal effects in them. In dogs, air was found in the arteries and left cavities of the heart only when death was delayed for some days after the experiment. This condition was found in three out of four cases, of the commission, thus delayed; in the fourth the heart and vessels were found collapsed and flabby, and containing no air, as was the case in all the similar experiments of Nysten. No distinction can be drawn between the post-mortem appearances in the cases where the admission of air was spontaneous and those where it was injected. Erichsen states that in many experiments by him the right cavities of the heart, though they contained bloody froth, were not distended by it (*op. cit.*, p. 8); and he draws an argument from this in favor of his theory, that distension of the heart has no thing to do with the production of death. However true the theory may be, the fact is entirely at variance with those remarked by other experimenters, as well as with the condition of the organ noticed in man after death from the same cause.

The brief *résumé* which we have now given of the results of experiment upon animals, imperfect though the experiments were, sufficiently establishes, by comparison with the accidents in man which we have considered, that the two classes are of the same nature, and due to the same cause.

Let us now consider another class of conditions, less understood than the preceding, under which air may enter the circulation and produce its characteristic effects, viz., through the veins of the uterus.

A fact illustrative of this possibility was observed by Legallois (*Jour. Hebd. de Méd.*, 1829, vol. iii. p. 183.) A female rabbit had had two successive inversions of the uterus after parturition, and he had placed himself to watch her, when all at once she struggled (*se débattre*) convulsively and died in less than three minutes. The right auricle was full of bubbles of air; the pulmonary artery, and the two anterior venæ cavæ contained them only in the portions near the heart, while the posterior cava was filled with them as far as the place where it received the uterine veins, and no farther. These veins themselves were also filled with air. M. Legâllois had seen the same thing in two other cases. His son, who relates this experience, asks if some of the cases of sudden death in women recently delivered, and where the autopsy has failed to disclose any adequate cause for the catastrophe, might not find explanation in this way; and M. Ollivier repeats the question, and pronounces it worthy of investigation (*op. cit.*). Some years later M. Nélaton observed that the liquid injected into the uterus of a woman who had died of erysipelas of the face, passed into and distended one of the veins of the large ligaments, driving before it bubbles of air (*Gazette Médicale*, 1840, p. 568).

In 1844, Prof. Simpson furnished Dr. John Reid with the following details, which first gave him the idea that death might occur from the introduction of air into the uterine veins. The patient had been delivered of twins; post-partum hemorrhage, with alternate contractions and relaxations of the uterus supervened, and she seemed to rally very imperfectly from the effects of the flooding. "I saw her," he says, "an hour or two afterwards. She had then a very weak and rapid, almost imperceptible pulse, an extremely anxious countenance, and here and there was an evanescent scarlatinoid rash over the surface of the body." After death the uterine and hypogastric-veins, and lower vena cava, as well as the larger veins of the extremities, were found full of frothy blood. He had since seen two other cases in which the same symptoms were present, but no autopsy was made.

Dr. John Rose Cormack read an essay on this branch of the subject before the Western Med. Society in 1850, but we have not access to his paper. It contained reports of seven cases, viz: that of Professor Simpson given above, one related by M. Bessems, particulars of which we shall give hereafter from the original source; and the five following, the few details of which we derive from the paper of Mr. May.

1st. A case by M. Lionet, of Corbeil: A lady, aged 27 years, had a natural labor with no hemorrhage. She soon became faint, breathed with difficulty, and expired five hours after delivery. Air was found in the heart and cerebral vessels. 2d. A case by Dr. Wirtrich: Convulsive movements and suffocation followed expulsion of infant and partial separation of placenta. Air was found in the venous system. The other three cases were observed by Dr. Lever, of Guy's Hospital. In all of them there was hemorrhage after delivery, and death in a few hours. Air was found in the uterine and other veins. The following are the important features of a case reported by Mr. Berry (*L. and E. Monthly Jour. of Med. Sci.*, 1851, p. 189, from *Prov. Med. & Surg. Jour.*). A lady, aged 22 years, was safely delivered of her first child about 7 o'clock P. M. The labor was natural, and there was no hemorrhage. She expressed herself as feeling comfortable up to 11 o'clock. At 1 o'clock her husband became alarmed by her difficult breathing and sent for the physician, but before he arrived, at 2 o'clock, she had expired. A post-mortem examination was made on the morning of the second day. The uterus was midway between the pelvis and umbilicus. The peritoneum covering it was healthy, but pale. The vessels where the placenta had been attached were patulous. The vagina contained, at its superior part, a moderately sized clot of blood. The heart was the size of a male heart, and apparently distended. Upon making an incision into it a gush of air escaped and the organ became flaccid. No blood was found in any of its cavities. The kidneys presented a granular appearance, and the lungs contained scattered tubercles in their upper lobes. The other organs were healthy, and there were no signs of decomposition.

The three following cases were reported by Mr. May in the *Br. Med. Journ.* (*Am. Journ. Med. Sci.*, Oct. 1857.) The first occurred to Mr. Taylor, of Wargrave. Labor was proceeding naturally when, as no urine had passed, he attempted to introduce a catheter. At this moment a severe pain occurred, accompanied by the escape of about $\frac{3}{4}$ pt. of liquor amnii. The patient exclaimed, "Oh! how faint I feel!" was convulsed a moment and expired. Autopsy 48 hours after death. Uterus above umbilicus. Placenta upon anterior surface, not separated. Scarcely any blood in uterus, lower vena cava, or heart. Right auricle thin and distended with air.

The second case was furnished by Mr. Smith, of White Church. The patient was naturally delivered, and comfortable. Severe after-pain occurred, and soon great oppression was felt about the chest, with sinking, exhaustion, and restlessness, speedily followed by death. Autopsy the same evening. Body not yet cold. The uterus was large, and contained a piece of the placenta adhering; it contained a coagulum, but not large enough to have produced death. The right side of the heart was distended. On opening it a puff of air escaped and the organ collapsed. The valves were healthy. A small clot was contained in the left ventricle.

The third case was seen by Mr. May himself. The labor had been natural and the patient had resumed her duties, when, on the eighth day after delivery, she was taken suddenly ill and expired. Autopsy the following day. No evidence of decomposition. Frothy blood was seen on slicing the liver. Air in inferior cava and venæ portæ. The right heart was distended with frothy blood. Uterus the size usual on the eighth day.

Professor Oppolzer relates a case of uterine carcinoma (*Br. and For. Med.-Chirurg. Rev.*, vol. xxx., 1862), in the course of which air entered the circulation. The attack began with acute pains in the hypogastrium, extending over right thorax. In about 24 hours the skin of the whole right side was intensely red, and covered with vesicles filled with reddish serum. The redness proved to be due to hemorrhage. A subcutaneous emphysema was observed in the epigastric region. Collapse set in, and death took place in a few hours. The details of the autopsy, which was made 33 hours after death, was very minute. The integments, as well as the spleen, crepitated on pressure. The viscera were pale and flaccid. Air or frothy blood was found in the right cavities of the heart, beneath the visceral layer of the pericardium, in the vessels of the liver and kidneys, the internal spermatic vein, and vena cava. The latter gave a tympanitic resonance on percussion. The disease proved to be villous cancer.

These eleven cases are all that we have been able to collect in which death was attributed to the spontaneous entrance of air through the uterine veins. Some of these are too meagre in detail to admit of an opinion being formed upon them; while in one or two it seems possible that the gas found in the vessels was developed there by morbid changes, but in other cases the evidence is strong in favor of the cause assigned, and when taken as a whole we think very little doubt can be entertained as to the nature of the accident. The chief obstacle to full belielf, in many cases, is the failure of the observers to state the precise condition of the body, as to post-mortem changes, and what precautions, if any, were taken to prevent the admission of air during the autopsy. But when we remember that the gentlemen who reported them were generally men of experience and eminent in the profession, and that the presence of air was suspected beforehand, we cannot doubt that, at least, ordinary precautions were taken against fallacy. It will be asked whether air may not be oftener present in the heart after death than is generally supposed, but only noticed when specially sought for. To this it may be answered that Dr. Cless, of Stuttguard, examined 1200 bodies after death from various diseases, with especial reference to this possibility, and only found air in fourteen cases, and those where the previous symptoms had led to the suspicion that air was present. (*Trans. of Path. Soc. of London*, vol. ix. p. 154.)

We now come to another class of cases, where the addition of another link in the chain of evidence renders it nearly or quite conclusive as to the cause of death. Among these we place the case of M. Bessems. It was one of adherent placenta in a hospital patient. Uterine injections of chlorinated water were being used, and previous to each injection small portions of the placenta had been torn away; the patient was weakened by loss of blood. It is stated that special care was taken to exclude bubbles of air from the syringe. On injecting the fluid for the third time, the woman immediately sprang up in bed (*se projette sur son séant*) and stretched out her arms, crying that she was suffocating. The head then fell backwards, the face grew pale, the eyes rolled upwards spasmodically (*se convulsent en haut*), and the look became fixed. It was thought to be an hysterical fit, but the respiration became less and less, the pulse was no longer perceptible, and, in spite of all the ordinary stimuli, death took place within three minutes after the injection. Autopsy twenty-four hours after death. No trace of putrefaction; the uterus somewhat larger than the fist; no sign of inflammation in its walls; its cavity still contained a portion of placenta of the size of a small hen's egg; the inferior cava was distended, and contained many large bubbles of gas distinctly visible through its walls; the right cavities of the heart were distended and elastic. After tying the vessels, the organ was opened under water, and a large quantity of gas mingled with blood escaped. The left cavities also contained some bubbles. In this case we cannot doubt that the gas found in the heart and vessels made its entrance at the time of the injection, and produced the fatal result. Whether the gas was the result of decomposition of the placenta, or was atmospheric air admitted from without, the effects would be the same, as proved by the experience of Nysten (*op. cit.*); but if it had been the former, fewer traces of it would probably have been discovered after death, on account of the great solubility of those gases in the blood.

Prof. Simpson relates an instance stated to him by an Irish practitioner. This gentleman was blowing common air into the os uteri for the purpose of procuring uterine contractions, when his patient suddenly fell back and expired, no bad symptoms having been previously observed. There was only very slight hemorrhage. We give this on the authority of Dr. A. D. Sinclair, of Boston.

Prof. Dalton relates another case (*Am. Med. Monthly*, June, 1860, p. 519), first reported by Dr. John Swinburne, of Albany. A female abortionist attempted to procure delivery at the fifth month by rupturing the membranes with a gutta-percha catheter, and the patient fell back, as the woman supposed, in a fainting fit; a physician was called, but before he arrived the patient was dead. Autopsy, in the month of March, fourteen hours after death. Air was found in the sinuses of the uterus, the jugular veins, and even in the superficial veins of the body. The right cavities of the heart were distended with a spumous mixture of blood and air. The uterus contained a healthy fœtus; the membranes were unbroken, but were separated from the uterine walls on the right side, together with a portion of the placenta, and there was a perforation at this spot leading directly in the uterine sinuses. Prof. Dalton coincides with Dr. S. in the opinion that air was forcibly blown in, through the catheter, with the intention of producing separation of the membranes. If this was the case, the way in which it entered the veins is sufficiently obvious.

The last case we have to record is the one which led us to examine the subject. It occurred under the observation of Dr. Homer O. Hitchcock, of Kalamazoo, Mich., and though not yet published, we are permitted by him to make use of the facts. An abortionist attempted to procure delivery of a woman four or five months pregnant, by perforating the membranes. He used a tube fourteen or fifteen inches long and about one-quarter inch diameter. Presently the husband, in the next room, heard his wife "screech," and a moment after the "doctor" opened the door and called for water, saying that the woman had fainted. The husband found her purple in the face, with the muscles of the chest and arms in contraction. Restoratives were tried to no purpose; she was dead in less than ten minutes. The man told the husband that the instrument did not hurt her until just as he was about to draw it out, when he blew in it, and she instantly struck at him with her hands, screamed out, and became blue in the face.

Viewed in connection with the facts and cases already considered, it would seem as if there could be no possible doubt as to the nature of the accident, even without the corroborative proof of a post-mortem examination; yet the physicians who conducted the autopsy had no suspicion of the presence of air in the vessels, and only examined the abdominal cavity and the uterus; while, if we mistake not, eminent surgical and pathological authority in this vicinity, on hearing the facts stated, expressed skepticism as to the agency of air in producing the result. Yet no less authority than Prof. Syme testified at the inquest in Dr. Willis's case, where death followed the puncture of a seton-needle, that an autopsy was not necessary to render certain the manner in which death was produced, and that it was only demanded by the interests of science. These gentlemen could not have been acquainted with the literature of the subject when expressing such an opinion. It only serves to illustrate the singular obscurity which veils the subject in this country, and the need there is that attention should be called towards it.

(*Concluded in next number.*)

